# Unveiling Post-COVID-19 syndrome: incidence, biomarkers, and clinical phenotypes in a Thai population

**DOI:** 10.1186/s12879-024-10055-2

**Published:** 2024-10-10

**Authors:** Sorawat Sangkaew, Hathaitip Tumviriyakul, Chutima Cheranakhorn, Nopporn Songumpai, Nawamin Pinpathomrat, Bunya Seeyankem, Kameelah Yasharad, Palanthorn Loomcharoen, Wisitsak Pakdee, Chanunya Changawej, Dararat Dumrongkullachart, Amornrat Limheng, Ilaria Dorigatti

**Affiliations:** 1 Department of Social Medicine, Hatyai Hospital, Songkhla, Thailand; 2 Department of Infectious Disease, Section of Adult Infectious Disease, Imperial College London. Hammersmith Hospital Campus, London, W12 0NN United Kingdom; 3 Department of Internal Medicine, Hatyai Hospital, Songkhla, Thailand; 4 Department of Biomedical Sciences and Biomedical Engineering, Faculty of Medicine, Prince of Songkla University, Songkhla, Thailand; 5 Office of Disease Prevention and Control Region 12, Songkhla, Thailand; 6 Department of Physical Medicine and Rehabilitation, Hatyai Hospital, Songkhla, Thailand; 7 Department of Radiology, Faculty of Medicine, Prince of Songkla University, Songkhla, Thailand; 8 Department of Nursing, Hatyai Hospital, Songkhla, Thailand; 9 Department of Community Nursing, Hatyai Hospital, Songkhla, Thailand; 10 MRC Centre for Global Infectious Disease Analysis, Department of Infectious Disease Epidemiology, School of Public Health, Imperial College London, London, UK

**Keywords:** Post-COVID-19 Syndrome, Long COVID-19, Biomarkers, Cluster analysis, Diagnosis, Clinical phenotypes

## Abstract

**Background:**

Post-COVID- 19 syndrome (PCS) significantly impacts the quality of life of survivors. There is, however, a lack of a standardized approach to PCS diagnosis and management. Our bidirectional cohort study aimed to estimate PCS incidence, identify risk factors and biomarkers, and classify clinical phenotypes for enhanced management to improve patient outcomes.

**Methods:**

A bidirectional prospective cohort study was conducted at five medical sites in Hatyai district in Songkhla Province, Thailand. Participants were randomly selected from among the survivors of COVID-19 aged≥18 years between May 15, 2022, and January 31, 2023. The selected participants underwent a scheduled outpatient visit for symptom and health assessments 12 to 16 weeks after the acute onset of infection, during which PCS was diagnosed and blood samples were collected for hematological, inflammatory, and serological tests. PCS was defined according to the World Health Organization criteria. Univariate and multiple logistic regression analyses were used to identify biomarkers associated with PCS. Moreover, three clustering methods (agglomerative hierarchical, divisive hierarchical, and K-means clustering) were applied, and internal validation metrics were used to determine clustering and similarities in phenotypes.

**Findings:**

A total of 300 survivors were enrolled in the study, 47% of whom developed PCS according to the World Health Organization (WHO) definition. In the sampled cohort, 66.3% were females, and 79.4% of them developed PCS (as compared to 54.7% of males, *p*-value <0.001). Comorbidities were present in 19% (57/300) of all patients, with 11% (18/159) in the group without PCS and 27.7% (39/141) in the group with PCS. The incidence of PCS varied depending on the criteria used and reached 13% when a quality of life indicator was added to the WHO definition. Common PCS symptoms were hair loss (22%) and fatigue (21%), while mental health symptoms were less frequent (insomnia 3%, depression 3%, anxiety 2%). According to our univariate analysis, we found significantly lower hematocrit and IgG levels and greater ALP levels in PCS patients than in patients who did not develop PCS (*p*-value < 0.05). According to our multivariable analysis, adjusted ALP levels remained a significant predictor of PCS (OR 1.02, *p*-value= 0.005). Clustering analysis revealed four groups characterized by severe clinical symptoms and mental health concerns (Cluster 1, 4%), moderate physical symptoms with predominant mental health issues (Cluster 2, 9%), moderate mental health issues with predominant physical symptoms (Cluster 3, 14%), and mild to no PCS (Cluster 4, 77%). The quality of life and ALP levels varied across the clusters.

**Interpretation:**

This study challenges the prevailing diagnostic criteria for PCS, emphasizing the need for a holistic approach that considers quality of life. The identification of ALP as a biomarker associated with PCS suggests that its monitoring could be used for early detection of the onset of PCS. Cluster analysis revealed four distinct clinical phenotypes characterized by different clinical symptoms and mental health concerns that 'exhibited varying impacts on quality of life. This finding suggested that accounting for the reduced quality of life in the definition of PCS could enhance its diagnosis and management and that moving toward personalized interventions could both improve patient outcomes and help reduce medicalization and optimally target the available resources.

**Funding:**

The research publication received funding support from Medical Council of Thailand (Police General Dr. Jongjate Aojanepong Foundation), Hatyai Hospital Charity and Wellcome Trust.

## Introduction

With more than 770 million infections and six million fatalities recorded, the global burden of coronavirus disease 2019 (COVID-19) is continuing to increase [1]. Beyond the acute phase of the illness, many survivors contend with post-COVID-19 syndrome (PCS), commonly known as long COVID-19. PCS is a multifaceted condition involving a wide array of persistent symptoms that affect individuals after an acute SARS-CoV-2 infection. Both the United Kingdom National Institute for Health and Care Excellence (NICE) [2] and the World Health Organization (WHO) [3] define PCS as the continuation or development of new symptoms 3 months following the initial infection, with symptoms persisting for at least 2 months and no alternative diagnosis.

In Thailand, the rapid spread of SARS-CoV-2 highlights the potential long-term burden of PCS within the population. The country experienced several waves of infection, most notably the Delta-driven fourth wave (July 15—December 15, 2021) and the Omicron-driven fifth wave (February 1—June 15, 2022). Although the Omicron wave resulted in higher hospitalization rates, the Delta wave was associated with greater severity and mortality. Understanding this local epidemiological landscape is essential for assessing the long-term health outcomes of COVID-19 survivors in Thailand and guiding strategies to address the impact of PCS.

The prevalence of PCS varies across studies due to differences in study populations and definitions. Recent meta-analyses suggest that PCS affects 42% (prediction interval = 6.8% to 87.9%) of COVID-19 survivors at the six-month postinfection mark [4, 5]. Notably, PCS significantly impairs individuals' quality of life, limiting their ability to work, study, and engage in daily activities [6–8].

Despite the high prevalence and impact of PCS on the quality of life of affected patients, a standardized approach to its diagnosis and management has not been established. This is partly attributable to the complex nature of the syndrome and the heterogeneity of its clinical presentation.

To address this knowledge gap, we conducted a bidirectional cohort study with the following objectives: estimating PCS incidence using both the conventional World Health Organization (WHO) diagnostic criteria [3] and a novel quality-of-life-based definition; identifying and characterizing PCS-associated risk factors and biomarkers for early detection and intervention; and classifying PCS clinical phenotypes to gain a deeper understanding of its diverse presentations.

## Methods

### Study design

A bidirectional prospective cohort study was conducted at five recruiting sites in a Hatyai regional hospital to cover all areas of the Hatyai district in Thailand. The study was approved by the Hatyai Hospital Ethics Committee (HYH EC 039–65-01).

### Participants

Eligible participants were randomly sampled from patients who had received care for COVID-19 at the regional hospital and survived 12 to 16 weeks after acute infection between May 15, 2022, and January 31, 2023. The inclusion criterion was individuals aged 18 years or older with laboratory evidence confirming COVID-19 infection through a positive rapid antigen test or RT–PCR test result.

### Procedures

A list of eligible participants was obtained from the hospital information system. The randomly sampled participants were contacted by phone 12 to 16 weeks after the onset of acute COVID-19 and provided with initial information about the study. Enrolled participants were scheduled for an outpatient visit.

Upon arrival at the scheduled outpatient visit, participants discussed the purpose, potential risks, and benefits of the study with a trained research nurse and provided written informed consent. The patients then underwent a history and physical examination to collect demographic and prior health status data (age, weight, height, body mass index, smoking status, alcohol consumption, comorbidities, vaccination, disease severity and treatment of acute COVID-19 infection, antiviral drugs). Participants engaged in validated assessments for key symptoms. The mMRC scale quantifies dyspnea [9], the Fatigue Score measures fatigue [10], the Mini-Cog charts cognitive function [11], the GAD-7 assesses anxiety [12], the Patient Health Questionnaire-2 and 9 gauge depression disorder [13], and the EQ-5D-5L captures overall quality of life [14]. Medical records were reviewed to obtain information on diagnosis and treatment during the acute phase. Vaccination data were obtained from the national vaccination registry.

A 10 ml venous blood sample was collected from all participants by trained research nurses. The following tests were performed on the blood samples at the regional hospital laboratory: complete blood count; liver function tests; blood urea nitrogen; creatinine; electrolytes; C-reactive protein; D-dimer; and lactate dehydrogenase. Interleukin-6 (IL-6) blood levels were tested at the Department of Biomedical Science, Faculty of Medicine, Prince of Songkla University. COVID-19 immunoglobulin G (IgG) level analysis was conducted at the Disease Control Medical Laboratory, Office of Disease Prevention and Control Region 12, and we assessed the anti-nucleocapsid protein (NCP) and the anti-receptor binding domain (RBD) of the S1 subunit of the spike protein. For the anti-NCP antibody analysis, we employed a semi-automated enzyme-linked immunosorbent assay (ELISA) on an I-2P Analyzer following the manufacturer's instructions. The anti-RBD antibody was detected using a fully automated chemiluminescent microparticle immunoassay (CMIA) on the ARCHITECT i System following the manufacturer’s instructions (Abbott Diagnostics, USA). This assay serves as a tool for evaluating the immune status of infected individuals and monitoring the antibody response in individuals who have received the COVID-19 vaccine by quantitatively measuring IgG antibodies against the spike RBD of SARS-CoV-2.

Chest radiographs, electrocardiograms, and thyroid function tests were considered for further visits based on clinical presentation and the clinician's judgment. The chest radiographs were reviewed by a radiologist with 5 years of experience to rule out alternative diagnoses for the presence of dyspnea. According to the World Health Organization, PCS is defined as the presence of any signs or symptoms that develop during or after infection lasting more than two months and that are not explained by an alternative diagnosis [15]. Patients who met the diagnostic criteria for post-COVID-19 syndrome were managed according to the hospital's standard of care.

### Statistical analysis

The data were transcribed from a paper-based case record form into Epidata (v3.2) with double data entry to ensure accuracy [16]. Any data mismatches were rechecked and corrected before analysis. All the data analyzed in this study were analyzed with the R program (version 4.0.2) [17]. Continuous data are presented as the mean or median with standard deviation or interquartile range, respectively, and categorical data are summarized as percentages. The normality of the continuous data was assessed using the Shapiro‒Wilk test. Independent t-tests were used to compare continuous data between patients with and without PCS if the data were normally distributed; otherwise, we used the Mann‒Whitney U test. Fisher's exact test was used to compare categorical data between patients with and without PCS. Univariate and multivariable logistic regression were used to evaluate the associations between factors and the occurrence of PSC. Odds ratios (ORs) with 95% confidence intervals (CIs) are presented. The 95% CI was calculated using the exact binomial CI for proportion outcomes. Multivariable logistic regression was performed, adjusting for age, sex, comorbidity, and the number of vaccine doses received, as these were predefined potential confounders. The selection of these variables was based on both clinical relevance and statistical significance from the univariate analyses. The area under the receiver operating characteristic curve (AUROC) was calculated to assess the discriminative value of each biomarker in predicting PCS. A *p*-value < 0.05 was considered to indicate statistical significance.

We used three unsupervised machine learning clustering methods (agglomerative hierarchical clustering, hierarchical divisive clustering, and K-means clustering) to explore the differences and similarities of clinical features collected among the patients. In agglomerative hierarchical clustering, each data point initially forms its own cluster, and the most similar clusters are iteratively merged. In hierarchical divisive clustering, all the data points initially belong to a single cluster, and the most dissimilar data points are iteratively split into separate clusters. In K-means clustering, the cluster centers are initially set using an agglomerative hierarchical algorithm, and the data points are assigned to the closest center. The cluster centers are then iteratively updated by minimizing the total sum of the squared errors of the distances between each data point and the closest centroid. This process is repeated until the cluster centers converge. We used the Euclidean distance as a similarity metric for all clustering methods and tested each method on multiple numbers of clusters (from 4 to 10). Ward's method was used for linkage in agglomerative hierarchical clustering.

## Results

### Characteristics of study participants

A total of 300 survivors of acute COVID-19 infection were enrolled in this study; these included both hospitalized and nonhospitalized patients. Among these participants, 47% (141/300) developed PCS, while the remaining 53% (159/300) did not (Table 1). The median age of the enrolled patients was 43 years, with an interquartile range (IQR) of 32–57 years. A total of 66.3% (199/300) of the participants were female, with a significantly greater proportion in the PCS group (79.4% (112/141) vs. 54.7% (87/159), *p*-value < 0.001). Comorbidities associated with COVID-19 severity were present in 19% (57/300) of the patients, with a greater prevalence among patients with PCS (27.7% (39/141) vs 11.3% (18/159), *p*-value < 0.001). Among all the study participants, 97.7% (293/300) received at least one vaccine dose, and individuals receiving a greater number of doses exhibited a significantly lower proportion of PCSs (3 and IQR 3–4 vs. 3 and IQR 2–3, *p*-value 0.003). The presence of PCS was lower among those receiving booster doses (79.9% (127/159) vs 69.5% (98/141)). The majority of the survivors (98.2%) received care in outpatient departments during the acute phase of the infection. Antiviral medications were administered to 57.3% (172/300) of the patients, followed by Favipiravir (37.3%), Molnupiravir (19.3%) and other antiviral drugs (0.7%).

**Table 1:** Baseline characteristics of study participants categorized by Post-COVID-19 Syndrome (PCS) status

Variables	Total	Post-Covid-19 Syndrome		*p*-value
		Absence	Presence	
Number (%)	300 (100)	159 (53)	141 (47)	
Age (y): median (IQR)	43.00 (32.0- 57.0)	40.00 (31.0- 57.0)	46.00 (33.0 56.0)	0.317
Age groups: number (%)				0.172
18–30	66 (22.0)	39 (24.5)	27 (19.1)	
32–45	100 (33.3)	57 (35.8)	43 (30.5)	
46–64	104 (34.7)	46 (28.9)	58 (41.1)	
≥ 65	30 (10.0)	17 (10.7)	13 (9.2)	
Female: number (%)	199 (66.3)	87 (54.7)	112 (79.4)	< 0.001
Male: number (%)	101 (33.7)	72 (45.3)	29 (20.6)	
BMI groups: number (%)				0.459
< 18	12 (4.0)	4 (2.5)	8 (5.7)	
18–24	160 (53.3)	84 (52.8)	76 (53.9)	
25–29	93 (31.0)	50 (31.4)	43 (30.5)	
≥ 30	35 (11.7)	21 (13.2)	14 (9.9)	
Comorbidity: number (%)	57 (19.0)	18 (11.3)	39 (27.7)	0.001
Cancer: number (%)	2 (0.7)	1 (0.6)	1 (0.7)	1.000
CVS: number (%)	13 (4.3)	4 (2.5)	9 (6.4)	0.175
CKD stage ≥ III: number (%)	5 (1.7)	2 (1.3)	3 (2.1)	0.892
Respiratory disease: number (%)	10 (3.3)	2 (1.3)	8 (5.7)	0.071
Neurological disease: number (%)	5 (1.7)	1 (0.6)	4 (2.8)	0.299
DM: number (%)	20 (6.7)	6 (3.8)	14 (9.9)	0.057
HIV: number (%)	3 (1.0)	0 (0.0)	3 (2.1)	0.205
Immunocompromised: number (%)	6 (2.0)	3 (1.9)	3 (2.1)	1.000
Vaccine doses: median (IQR)	3 (3- 4)	3 (3- 4)	3(2- 3)	0.003
Vaccine course: number (%)				0.054
unvaccinated	7 (2.3)	1 (0.6)	6 (4.3)	
incomplete	1 (0.3)	0 (0.0)	1 (0.7)	
complete	67 (22.3)	31 (19.5)	36 (25.5)	
boosted	225 (75.0)	127 (79.9)	98 (69.5)	
mRNA vaccine doses: median (IQR)	1.00 (1.00- 2.00)	1.00 (1.00- 2.00)	1.00 (1.00- 2.00)	0.178
Acute phase				
Hospitalization: number (%)	5 (1.7)	1 (0.6)	4 (2.8)	0.299
Receiving antiviral drugs: number (%)				0.179
No	128 (42.7)	66 (41.5)	62 (44.0)	
Favipiravir	112 (37.3)	66 (41.5)	46 (32.6)	
Molnupiravir	58 (19.3)	27 (17.0)	31 (22.0)	
Remdesivir	2 (0.7)	0 (0.0)	2 (1.4)	
Oxygen therapy: number (%)	2 (0.7)	0 (0.0)	2 (1.4)	0.426
ICU admission: number (%)	2 (0.7)	0 (0.0)	2 (1.4)	0.426

### Incidence and clinical features of Post-COVID Syndrome (PCS)

**Fig. 1:**
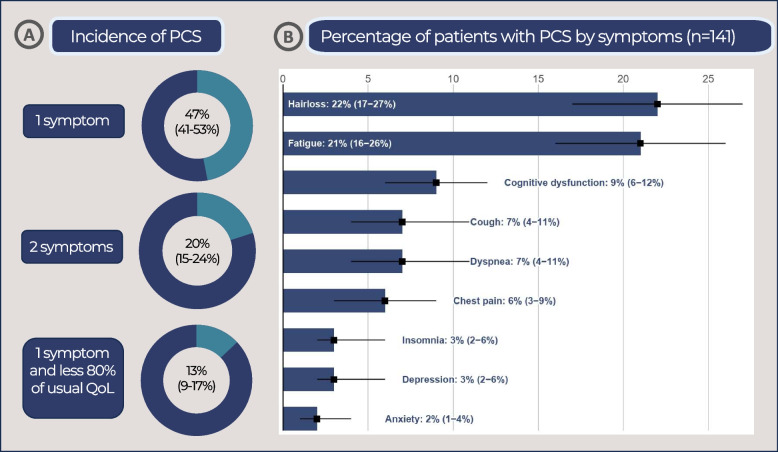
Incidence of post-COVID-19 syndrome (PCS) based on clinical symptoms, quality of life, and patient-reported symptoms among PCS patients. (QoL; quality of life, PCS: post-COVID-19 syndrome)

Figure 1 shows the incidence of PCS according to different criteria (A) and the number of patients with PCS categorized by the symptoms they presented (B). Based on the WHO criteria, which consider the persistence of any symptom for more than two months following recovery from acute COVID-19 for three months, the incidence of PCS was approximately 47% (95% confidence interval (CI): 41%-53%). When PCS was defined as the presence of at least two persistent symptoms, the incidence decreased to 20% (95% CI 15%-24%). The inclusion of quality-of-life impairment, defined as less than 80% of usual health status, as a criterion further reduced the incidence of PCS to 13% (95% CI 9%-17%).

Among patients with PCS, the most common symptoms were hair loss (mean 22%, 95% CI 17%-27%), fatigue (mean 21%, 95% CI 16–26%), and cognitive dysfunction (mean 9%, 95% CI 6–12%). Other reported symptoms included cough (mean 7%, 95% CI 4–11%), dyspnea (mean 7%, 95% CI 4–11%), and chest pain (mean 6%, 95% CI 3–9%). Less frequent symptoms affecting mental function included insomnia (mean 3%, 95% CI 2–6%), depression (mean 3%, 95% CI 2–6%), and anxiety (mean 2%, 95% CI 1–4%).

Table 2 shows female sex was significantly associated with PCS, with an adjusted odds ratio (OR) of 3.51 (95% CI: 2.06–6.12, *p* < 0.001). The number of underlying diseases was also positively associated with the outcome (adjusted OR: 2.98, 95% CI: 1.55–5.93, *p* = 0.001). In contrast, the number of vaccine doses is inversely associated with PCS (adjusted OR: 0.66, 95% CI: 0.49–0.86, *p* = 0.003).

**Table 2:** Association between biomarkers and post-COVID syndrome examined through univariate (crude) and multiple (adjusted) logistic regression analyses

Biomarkers	Crude ORs (95% CI)	*p*-value	Adjusted ORs (95% CI)	*p*-value	AUROC (95% CI)
**Demographic and Clinical Characteristics**					
Age (year)	1.01 (0.99–1.02)	0.350	1 (0.99–1.02)	0.607	
Female	3.20 (1.93–5.40)	** < 0.001**	3.51 (2.06–6.12)	** < 0.001**	
BMI (kg/m^2^)	0.97 (0.92–1.02)	0.200	0.97 (0.92–1.03)	0.326	
Number of underlying diseases	3 (1.64–5.64)	** < 0.001**	2.98 (1.55–5.93)	**0.001**	
Vaccine doses	0.65 (0.49–0.84)	** < 0.001**	0.66 (0.49–0.86)	**0.003**	
**Laboratory**					
HCT	**0.91 (0.85–0.96)**	**0.001**	0.98 (0.91–1.06)	0.605	0.61 (0.55–0.68)
WBC	1.03 (0.91–1.17)	0.663	1.03 (0.9–1.18)	0.705	0.51 (0.44–0.57)
PLT	1 (1–1)	0.909	1 (1–1)	0.741	0.53 (0.46–0.53)
NLR	0.95 (0.72–1.23)	0.691	0.93 (0.7–1.24)	0.611	0.55 (0.49–0.62)
PLR	1 (0.99–1)	0.260	1 (0.99–1)	0.194	0.52 (0.45–0.58)
AST	1 (0.97–1.03)	0.803	1.01 (0.98–1.04)	0.652	0.48 (0.42–0.55)
ALT	1 (0.99–1.01)	0.805	1.01 (1–1.03)	0.286	0.47 (0.40–0.54)
AST/ALT ratio	1.18 (0.72–1.95)	0.501	0.85 (0.49–1.46)	0.557	0.53 (0.46–0.59)
ALP	**1.02 (1.01–1.03)**	**0.005**	**1.02 (1–1.03)**	**0.016**	0.61 (0.55–0.68)
Albumin	0.55 (0.25–1.18)	0.128	1.26 (0.5–3.2)	0.628	0.56 (0.50–0.63)
CRP	1.02 (0.97–1.08)	0.404	1.03 (0.98–1.09)	0.303	0.52 (0.47–0.60)
D-dimers	1 (1–1)	0.364	1 (1–1)	0.677	0.53 (0.46–0.60)
IL-6	1 (0.98–1.01)	0.418	0.99 (0.98–1.01)	0.340	0.51 (0.44–0.57)
log (IgG)	**0.78 (0.62–0.96)**	**0.024**	0.84 (0.64–1.09)	0.203	0.57 (0.50–0.64)

Mutlivariable logistic regression was adjusted for age, sex, comorbidity, and the number of received vaccine doses

#### Biomarkers for Diagnosing Post-COVID Syndrome

Biomarkers measured between three and four months after the acute phase were analyzed to aid in the clinical diagnosis of PCS (Table 2). A lower hematocrit level (OR 0.91, 95% CI 0.85–0.96) and lower IgG level (OR 0.78, 95% CI 0.62–0.96), as determined by univariate logistic regression analyses, were found to be associated with the occurrence of PSC. Conversely, elevated levels of alkaline phosphatase (ALP) (OR 1.02, 95% CI 1.01–1.03) were identified as a factor linked to PSC diagnosis in the univariate models. After adjusting for age, sex, comorbidities, and vaccination status in multivariate models, higher ALP levels remained significantly associated with PCS diagnosis, with an OR of 1.02 (95% CI 1–1.03). The area under the receiver operating characteristic curve (AUROC) for ALP was 0.61 (95% CI 0.55–0.68). Other hematological and inflammatory biomarkers, including CRP, interleukin-6, and D-dimer levels, were not significantly associated with PCS.

### Clinical phenotypes in post-covid syndrome survivors by clustering analysis

**Fig. 2:**
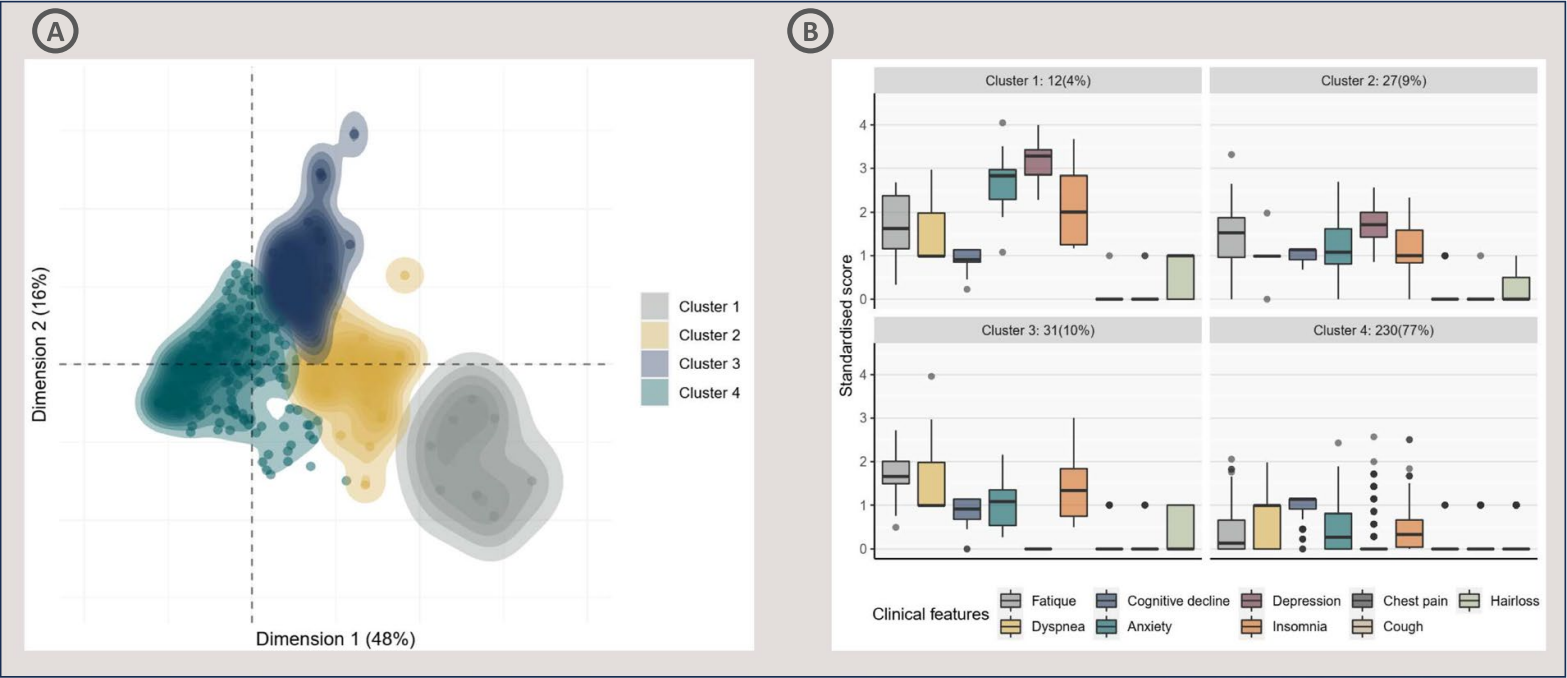
**(A)** Divisive hierarchical clustering reveals four distinct clusters among 300 survivors after 3 months. The x- and y-axes represent the two principal components from principal component analysis and account for 48% and 16%, respectively, of the variance in the data. The color shading represents the density of the data, and (**B**) shows the clinical phenotypes associated with each cluster. Medians (bold lines), interquartile ranges (boxes), ranges (vertical lines) and outliers (points) of clinical scores characterizing the four clusters obtained via divided hierarchical clustering

Employing divisive hierarchical clustering yielded the best clustering solution, as it achieved the highest weighted aggregate score, and revealed four distinct clusters, as depicted in Fig. 2a. The positioning of each data point (representing an individual subject) within the clusters was interpreted as the clinical phenotype employed as a variable in the analysis. In Fig. 2b, the clinical scores of symptoms for survivors are delineated within each cluster. Cluster 1, characterized by severe symptoms and mental health concerns (12 subjects, 4%), encompasses patients with high scores across multiple clinical features, with a predominance of mental health-related issues. Cluster 2, representing moderate symptoms and mental health concerns (27 subjects, 9%), included patients with moderate scores across various clinical features, primarily related to mental health issues. Cluster 3, characterized by moderate physical symptoms (31 subjects, 10%), included patients with moderate clinical features, predominantly characterized by physical limitations. Cluster 4, representing mild to no PCS (230 subjects, 77%), encompasses patients with minimal clinical symptoms or no PCS symptoms. This cluster constituted the majority of the study population.

**Fig. 3:**
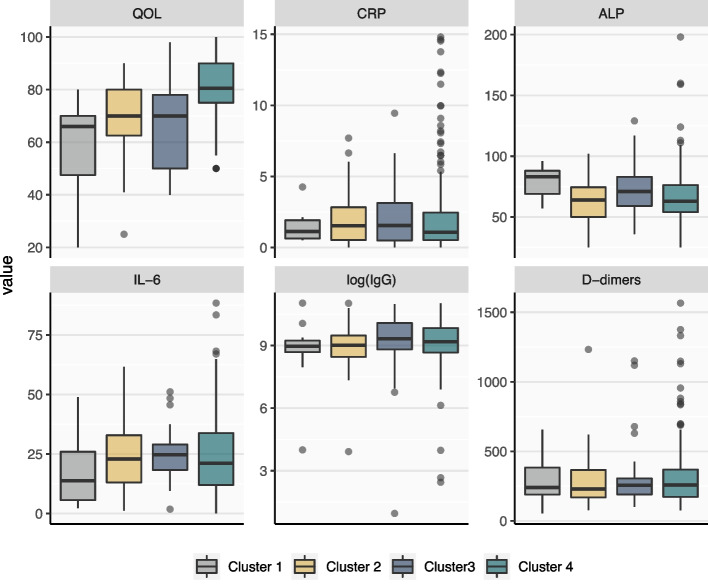
Quality of life assessment and biomarker levels across the identified clusters (QOL: quality of life; CRP: c-reactive protein; ALP: alkaline phosphatase; IL-6: interleukin-6)

The four clusters exhibited significant differences in quality of life (Fig. 3). Cluster 1, characterized by severe symptoms across multiple clinical features, demonstrated the lowest quality of life. Clusters 2 and 3, representing patients with moderate mental health issues and physical fatigue, respectively, exhibited intermediate quality of life. In contrast, Cluster 4, comprising patients with minimal clinical symptoms, had the highest quality of life compared to the other clusters. The average levels of ALP were the highest and lowest in Cluster 1 and Cluster 4, respectively. No obvious trends were observed for the other biomarkers.

## Discussion

This study estimated the incidence of PCS among patients who recovered from acute COVID-19 infection three months after initial infection, identified biomarkers associated with PCS diagnosis, and identified four clusters of patients who recovered from acute COVID-19 infection based on their clinical phenotypes. The findings also revealed relationships between quality of life and PCS-associated biomarkers.

This study revealed a 47% incidence of PCS among patients with a diagnosis of COVID-19 infection presenting at Hatyai Hospital in the Songkhla Province of Thailand, which is consistent with the findings of previous meta-analyses [4, 5, 18]. Twelve percent of these patients reported an impaired quality of life lower than 80% of their usual health status. These findings suggest that clinical and mental health interventions may be most needed for this subset of patients and could help target resource allocation and reduce unnecessary medical interventions. The incorporation of quality-of-life assessment into the PCS diagnostic criteria could help identify patients who require clinical, physical and mental health attention, which could minimize the burden on healthcare systems and reduce medicalization.

Our study revealed that post-COVID-19 symptoms, including hair loss, fatigue, and cognitive dysfunctions, align with the literature [4, 18]. Notably, the greater occurrence of hair loss than of fatigue in the present study is likely influenced by the fact that the group of patients was mostly female and that a significant proportion of patients exhibited mild acute-phase severity [18–20]. While persistent symptoms such as cough, dyspnea, and chest pain are prevalent in PCS patients, an increase in PCS-related mental health symptoms, including insomnia, anxiety, and depression, underscores the crucial role of multidisciplinary care teams in providing comprehensive care for individuals in the post-COVID-19 phase, promoting the restoration of their quality of life [21].

Our investigation identified biomarkers associated with PCS, including elevated ALP levels and decreased HCT and immunoglobulin G (IgG) levels. Elevated ALP levels suggest a connection with the inflammatory process, as ALP is an established inflammatory biomarker [22, 23], although the utility of ALP in aiding diagnosis is limited due to its low discriminative performance.

Cluster analysis revealed diverse clinical phenotypes among post-COVID-19 survivors, paving the way for targeted interventions. Patients in Cluster 1 exhibited multiple symptoms and had the lowest quality of life scores. These individuals face a complex array of physical and mental health challenges, demanding heightened attention and comprehensive health services. A multidisciplinary approach encompassing specialists across various disciplines is crucial for managing diverse symptoms and improving overall well-being. Previous studies have confirmed these findings [24, 25]. Research has identified a similar cluster characterized by a multi-symptom burden encompassing both physical and mental health concerns. Cluster 1 consistently demonstrated a greater impact on general health status and workability, highlighting the severity of this phenotype [25]. Clusters 2 and 3 presented with moderate, multi-symptomatic profiles, suggesting further nuance to the landscape of post-COVID syndrome. Cluster 3 progresses toward a predominance of physical symptoms characterized by fatigue, dyspnea, and other bodily manifestations. In contrast, Cluster 2 exhibited a predominance of mental health symptoms, where anxiety, depression, and other psychological challenges were at the center stage. These findings echo previous research from Canada, which also identified two distinct recovery groups: one dominated by physical symptoms and another dominated by mental health concerns [24]. This congruence underscores the heterogeneity within the umbrella of post-COVID syndrome research. While some individuals grapple with predominantly physical manifestations, others face the battle with mental health challenges, and a subset experiences a complex interplay of both. Recognizing these distinct presentations is crucial for tailoring appropriate interventions and for providing targeted support for each cluster. Reassurance and regular monitoring may suffice for managing any lingering symptoms and encouraging healthy habits for Cluster 4. This strategy can help avoid overburdening healthcare systems.

This study represents an inaugural cohort study of PCSs in Thailand involving the follow-up of randomly selected survivors. Routine biomarkers were assessed three months post-recovery to identify diagnostic aids. Utilizing machine learning, we clustered patients into PCS phenotypes, contributing to a deeper understanding of PCS and facilitating more equitable, personalized and comprehensive care in Thailand and similar healthcare contexts in the region. This study has several limitations, as it involved recruiting patients with predominantly mild symptoms from the outpatient department, leading to limited statistical power for factors related to severity, such as steroid use, oxygen therapy, or antiviral medication. The sample may not be representative of the broader population of COVID-19 survivors, particularly those with moderate to severe symptoms who may experience different clinical outcomes. Additionally, the study lacked comprehensive information on the symptoms and mental health status during the acute phase, preventing the evaluation of changes from the acute phase, and the cognitive assessment was not detailed enough to detect brain fog syndrome or cognitive impairment. There are also limitations in self-reported data on symptoms and mental health, leading to potential recall bias that could affect the accuracy of the information collected. Lastly, the follow-up period may be insufficient to capture the full spectrum of PCS, as symptoms can evolve, and longer-term effects may not be fully assessed.

Our study, based on a cohort of COVID-19 survivors in Thailand, provides valuable insights into PCS incidence, biomarkers, and clinical phenotypes. However, the generalizability of these findings may be limited by differences in ethnic, genetic, and sociodemographic characteristics, as well as healthcare systems, compared to other countries. Our identification of biomarkers, particularly ALP, as a possible predictor of PCS, offers a potential framework for other populations, though further studies are needed to validate these biomarkers in different clinical and geographical contexts. Importantly, the clustering analysis revealing distinct patient phenotypes has broad clinical implications, suggesting that personalized care pathways based on symptom severity and quality of life considerations could be beneficial across healthcare settings globally. This approach aligns with the growing emphasis on precision medicine and multidisciplinary care in post-COVID-19 management. Incorporating quality-of-life indicators into PCS definitions supports a more holistic, patient-centered approach, aiding clinicians in refining diagnosis and management strategies across various healthcare contexts.

In conclusion, our pioneering study on PCS in Thailand provides crucial insights into the incidence of PCS, diagnostic markers, and characteristics of patient clusters. The inclusion of quality-of-life assessments in the diagnosis of PCS and long COVID-19 could help identify patients who are at increased need of further investigation and comprehensive care, thus helping optimize resource allocation and reduce unnecessary interventions. While biomarkers show associations with PCS, their limited diagnostic performance necessitates further investigation. The identification of distinct patient clusters emphasizes the importance of tailored interventions, highlighting the role of multidisciplinary care teams in comprehensive post-COVID-19 management. These findings offer a globally relevant framework for PCS studies, contributing to informed healthcare policies, resource allocation, and targeted interventions for the well-being of post-COVID-19 survivors in diverse contexts.

### Panel: Research in context

#### Evidence before this study

Previous studies suggest that following the acute phase of Coronavirus Disease 2019 (COVID-19), nearly half of survivors, regardless of hospitalization, experience its long-term consequences, known as Post-COVID-19 Syndrome (PCS) or long COVID. However, existing diagnostic criteria primarily consider physical symptoms, potentially overlooking the significant impact on quality of life and daily functioning. This approach has the potential to result in unnecessary medical interventions and places an increased burden on global public health systems. Although researchers have explored various biomarkers for PCS, none have definitively established diagnostic evidence, contributing to growing laboratory costs. Furthermore, the effective treatment of PCS remains elusive, and the lack of comprehensive care models involving multidisciplinary teams adds complexity to managing this syndrome.

#### Added value of this study

This study suggests that the inclusion of changes in the quality of life in the diagnostic criteria PCS could offer a more holistic approach to the identification of long COVID, and help reduce unnecessary medical interventions overall, given patients have been found to suffer from a complex combination of physical symptoms and mental health concerns which affect their quality of life differently. Additionally, it identifies Alkaline Phosphatase (ALP) as a biomarker with a potential association with PCS three months after the acute phase, showcasing promise for earlier diagnosis compared to other biomarkers. Notably, the study's cluster analysis unveils four distinct phenotypes among post-COVID-19 survivors, paving the way for more personalized treatment plans. These findings hold significant promise for improving both diagnosis and management of PCS, ultimately leading to better patient outcomes.

#### Implications of all the available evidence

The findings of this study carry significant implications for the understanding and management of PCS. By underscoring the limitations of prevailing diagnostic criteria, which predominantly focus on physical symptoms, the research advocates for a more holistic approach by considering the impact on individuals' quality of life. This shift has the potential to mitigate unnecessary medical interventions, thereby reducing the strain on global public health systems. The identification of ALP as a prospective biomarker associated with PCS suggests the possibility of an earlier diagnosis, although further research is necessary for refinement. Moreover, the delineation of four distinct clinical phenotypes through cluster analysis provides a framework for moving toward personalized treatment plans, offering tailored interventions for specific manifestations. These insights not only guide comprehensive specialist care but also suggest targeted approaches such as physical therapy or mental health support based on individual symptomatology. Overall, these findings offer a promising avenue for enhancing the diagnosis and management of PCS, ultimately aiming for improved patient outcomes.

## Data Availability

The datasets utilized and/or analyzed during the current study are available from the corresponding author upon reasonable request.
